# Lanthanide-based β-Tricalcium Phosphate Upconversion Nanoparticles as an Effective Theranostic Nonviral Vectors for Image-Guided Gene Therapy

**DOI:** 10.7150/ntno.68789

**Published:** 2022-02-16

**Authors:** Flavia Rodrigues Oliveira Silva, Nelson Batista Lima, Maria Helena Bellini, Luiz Felipe Silva Teixeira, Eric Yiwei Du, Niloufar Jamshidi, Justin Gooding, Adam David Martin, Alexander Macmillan, Christopher Peter Marquis, Pall Thordarson

**Affiliations:** 1 Instituto de Pesquisas Energéticas e Nucleares, São Paulo, 05508-000, Brazil; 2 School of Chemistry, Faculty of Science, The University of New South Wales, Sydney NSW 2052, Australia; 3 School of Biotechnology and Biomolecular Sciences, Faculty of Science, The University of New South Wales, Sydney NSW 2052, Australia; 4 Dementia Research Centre, Department of Biomedical Sciences, Macquarie University, Sydney, NSW 2019, Australia; 5 Katharina Gaus Light Microscopy Facility, Mark Wainwright Analytical Centre, Lowy Cancer Research Centre, The University of New South Wales, Sydney, NSW 2052, Australia; 6 The UNSW RNA Institute, The University of New South Wales, Sydney NSW 2052, Australia

**Keywords:** lanthanide-based β-TCP, NIR upconversion theranostic nanoparticles, PEI, TAT, gene therapy.

## Abstract

Lanthanide-based beta-tricalcium phosphate (β-TCP) upconversion nanoparticles are exploited as a non-viral vector for imaging guided-gene therapy by virtue of their unique optical properties and multi-modality imaging ability, high transfection efficiency, high biocompatibility, dispersibility, simplicity of synthesis and surface modification. Ytterbium and thulium-doped β-TCP nanoparticles (βTCPYbTm) are synthesized via co-precipitation method, coated with polyethylenimine (PEI) and functionalized with a nuclear-targeting peptide (TAT). Further, *in vitro* studies revealed that the nanotheranostic carriers are able to transfect cells with the plasmid eGFP at a high efficiency, with approximately 60% of total cells producing the fluorescent green protein. The optimized protocol developed comprises the most efficient βTCPYbTm/PEI configuration, the amount and the order of assembly of βTCPYbTm:PEI, TAT, plasmid DNA and the culturing conditions. With having excellent dispersibility and high chemical affinity toward nucleic acid, calcium ions released from βTCPYbTm:PEI nanoparticles can participate in delivering nucleic acids and other therapeutic molecules, overcoming the nuclear barriers and improving the transfection efficacy. Equally important, the feasibility of the upconversion multifunctional nanovector to serve as an effective contrast agent for imaging modality, capable of converting low-energy light to higher-energy photons via a multi-photons mechanism, endowing greater unique luminescent properties, was successfully demonstrated.

## Introduction

Theranostic agents have emerged as a powerful multifunctional modality to integrate therapeutic and diagnostic strategies in a single all-in-one particle. Optical imaging approaches are promising high-resolution modalities for cellular and molecular imaging, for noninvasively self-reporting in real-time to monitor uptake and drug/gene delivery 1. In the case of theranostic nanoparticles 2-4, when combined with either their intrinsic therapeutic properties or the incorporation of a therapeutic molecule into nanoparticles, they can be used as a light-controlled drug, as photosensitizers, and therapeutic delivery systems, including genes 5.

In the past few decades, advances in the field of the nanomedicine and nanotechnology have had considerable impact on the development of novel nanoparticle-based probes for *in vivo* and *in vitro* cancer molecular imaging optical sensitivity contrast agents in the visible and the near-infrared spectrum (NIR, typically in the 700-1300 nm spectral range). At the NIR spectrum window, biological tissues show very low absorption and subsequently low autofluorescence 5,6. As a result, the sensitivity of NIR imaging contrast agents is greatly enhanced over visible light-based imaging agents, affording deeper penetration than that of visible light, and low phototoxicity to normal cells and tissues 7.

In applications where long-time studies or extended illumination is required, the greater photostability of inorganic upconverting nanoparticles as NIR fluorescence probes over organic fluorophores has generated interest in this class of optically active nanoparticle 7. A new class of lanthanide-based beta-tricalcium phosphate nanoparticles (βTCPYbTm) was recently developed that has been shown to possess an efficient upconversion process involving the Yb^3+^ → Tm^3+^ energy transfer to produce visible emissions from the ^1^G_4_ and ^3^F_2_ excited states of Tm^3+^ in the blue (478 nm, with a luminescence decay time of 153 μs), and red (647 nm, decay time of 120 μs) spectral emissions, respectively, and NIR emission from the ^3^H_4_ level (800 nm), under laser excitation centered in the Yb^3+^ ions absorption at 973 nm 8.

The βTCPYbTm nanoparticles possess several favorable properties including upconversion luminescence, large anti-Stokes shift, NIR absorption spectra, sharp emission bands, long excited-state decay lifetimes, non-photobleaching, lower interference from cells and tissues autofluorescence, and high biodegradability. Nanoparticle complexes comprised of βTCPYbTm and genetic material (DNA or RNA) have thus been proposed as a multifunctional nanocarriers (nanocomplexes) for gene therapy.

Gene therapies can potentially be used for the treatment and prevention of a variety of diseases. It is a technique based on the transfection of functional genes to replace or silence defective genetic material 1,5. The major challenge of gene therapy is the successful delivery of the nucleic acid into the targeted cell nuclei. Different carriers have been investigated: viral vectors, naked DNA and nonviral vectors 9. Viral vectors are by far the most studied, despite achieving higher efficiencies, their costly/complex production and the fact that they may not be well suited to all cellular targets are their major limitations. Naked DNA requires electroporation which can be very damaging to target cells and is mainly used in *ex vivo* settings. Synthetic nonviral vectors, such as nanoparticles, are increasingly being considered as an attractive alternative 10.

One of the earliest gene transfection methods with inorganic carrier involves the mixing of DNA with calcium chloride and phosphate containing buffer, to form CaP precipitates carrying DNA 11, which was firstly introduced by Graham in 1973 10. However, although it has been used as a routine laboratory procedure, this genetic method does not show good reproducibility among *in vitro* experiments. This is because once CaP-DNA precipitates, the size and shape of the nanoparticles is hard to control and therefore the CaP vectors suffer from variation in their efficacy 11.

Calcium phosphate (CaP) nanoparticles, in the form of amorphous phase and hydroxyapatite (HA) have demonstrated high efficiency and biocompatibility when compared with other nonviral gene carriers 9,12. Calcium ions are known to play an important role in gene transfection, forming ionic compounds with phosphates of DNA double helix, protecting the gene from degradation, and facilitating the delivery of nucleic acid into the nucleus across the cell membrane via ionic-channel mediated endocytosis of the target cell 11. In this context, β-tricalcium phosphate (β-TCP) would be advantageous over HA, since the former presents a higher solubility rate 13, leading to more calcium free ions, which, under such circumstances, would form the DNA-Ca^2+^ complexes and easily enter the nucleus through the nuclear pore complexes (NPC). In addition, CaP nanoparticles can be coated with desirable biomolecules (*e.g.*, antibodies, peptides, polymers, etc) to facilitate the complexation with the gene and/or to provide targeting and selectivity to specific cells.

In this work, the synthesis of a novel nanocomplex of a polyethylenimine (PEI)-coated lanthanide-based β-TCP and/or synergistically functionalized with nuclear targeting peptide (TAT, Trans-Activator of Transcription, generated from the human immunodeficiency virus 1) 14 is described. Subsequently, these particles were tested for cellular gene transfection to evaluate their use as efficient and biocompatible nonviral vectors for image-guided gene therapy.

## Experimental Section

### Synthesis and characterization of βTCPYbTm nanoparticles

The βTCPYbTm nanoparticles were synthesized by a co-precipitation method, wherein the phosphoric acid (H_3_PO_4_, Synth - Brazil) diluted in MilliQ water (0.3 M) was slowly added drop by drop (8 mL min^-1^ rate) into a suspension of calcium hydroxide (Ca(OH)_2_, Synth- Brazil), ytterbium nitrate (5.5 %, Sigma Aldrich) and thulium nitrate (0.5 %, Sigma Aldrich) (0.5 M) in MilliQ water. The initial (Ca+Yb+Tm)/P molar ratio was fixed at 1.5. The pH value of the solution was monitored to ensure it remained below 6 during the precipitation process. The precipitates were filtered and washed several times with deionized water to remove free ions, and re-dispersed in ethanol and dried at 60°C for 24 h. Finally, the powder was thermally treated at 900 °C for 3 min, in a specially adapted domestic microwave oven.

The nanoparticles were characterized by X-ray diffraction (XRD) using a Multiflex Rigaku diffractometer and the Cu kα (λ = 0.1542 nm) radiation. The nanopowder was dispersed in deionized water, using an ultrasonic bath, and then dropped on a copper grid to be observed by transmission electronic microscopy (TEM, JEM 2100 - JEOL).

In the luminescence measurements, the samples were excited by pulsed laser radiation generated by a tunable OPO-IR pumped (Rainbow from OPOTEK, USA) by the second harmonic of a Q-switched Nd:YAG (yttrium aluminum garnet) laser (BrilliantB from Quantel, France). Laser pulse widths of 4 ns at 973 nm were used to directly excite the ^4^F_5/2_ excited state of Yb^3+^. Luminescence signals were analyzed by the 0.25 m Kratos monochromator, detected by a charge coupled device (CCD) spectrometer coupled to the sample holder containing the nanopowder via optical fiber.

### PEI-functionalized βTCPYbTm nanoparticles

Polyethylenimine (PEI, branched, Mw 25.000 g.mol^-1^, from Sigma-Aldrich)-functionalized βTCPYbTm nanoparticles (βTCPYbTm:PEI) were prepared as follows: First, a stock solution of 0.1 mg mL^-1^ of PEI in MilliQ water (Millipore), with pH adjusted to 7.2-7.4 with HCl, was produced and kept at 4 °C. Then, different amounts of calcium phosphate nanoparticles, varying from 0.1 to 10.0 mg, corresponding to 10 to 1000 ng mol^-1^, were mixed with 10 mL of PEI solution, for a CaP/PEI ratio from 12.5 to 250:1 w/w, and sonicated for 20 min in an ultrasonic water bath (Unisonic FXD12 50 Hz). The final pHs of the suspensions were between 7.1 and 7.3, without further adjustment. Suspension of the calcium nanoparticles without the addition of PEI was also prepared similarly as described above and designated as bare βTCPYbTm.

The size and surface charge of fresh PEI-functionalized βTCPYbTm nanoparticles were evaluated by DLS analysis (Zetasizer Nano ZS - Malvern Panalytical).

### Formation of βTCPYbTm:PEI/pDNA complexes

The plasmid (pDNA) pEGFP-C1 (Clontech), containing the gene encoding the enhanced green fluorescent protein reporter driven by the CMV promoter, was purified from JM109 transfected cells using the GenElute TM HP Plasmid Midiprep Kit (Sigma) according to the manufacturer's instructions.

The ability of βTCPYbTm:PEI complexes to bind DNA at different nanoparticles: polymer ratios was investigated by a gel mobility retardation assay: complex solutions (30 μL) at different weight ratios were prepared freshly as described above and were mixed with pDNA (300 ng) and vortexed for 3 seconds. After incubation for 20 min at room temperature, the resultant dispersions of complexes (15 μL) containing the pDNA were mixed with loading buffer (5 μL), and loaded on an agarose gel (1.2 %, E-Gel with Sybr safe - Invitrogen). The electrophoresis experiments were carried out at 100 V for 26 min and the pDNA bands were visualized using the E-Gel power snap electrophoresis system from Invitrogen.

### TAT- nanocomplexes

The TAT (48-60) peptide, sequence [(Gly)(Arg)(Lys)(Lys)(Arg)(Arg)(Gln)(Arg)(Arg)(Arg)(Pro)(Pro)(Gln) - GRKKRRQRRRPPQ] was purchased from GenScript (USA). TAT (48-60) was reported to be the most active peptide from different TAT sequences 15. In order to evaluate TAT internalization, it was also purchased labeled with the TAMRA fluorophore.

For TAT / TAT-TAMRA solutions, the peptides were dissolved in PBS (1 mg mL^-1^) and passed through a 0.22 μm pore size filter, stored in sterile 1.5 mL tubes and kept at -20 °C. The TAT-TAMRA solution was also kept protected from light.

### Cell transfection (optimized protocol)

The gene transfection experiments were performed in human embryonic kidney 293 fast-growing (HEK293T) cells using the plasmid eGFP-C1 (Addgene / Clonatech) as the exogenous reporter gene.

Cells were maintained in DMEM medium (supplemented with 10% fetal bovine serum (FBS), 1% penicillin and 1% streptomycin) at 37 °C and 5% CO_2_. A day prior to transfection, HEK293T cells were seeded in a 12-well plate at a concentration of 10^5^ cells per well. On the day of transfection, when cells were 70-80 % confluent, the βTCPYbTm:PEI-pDNA or βTCPYbTm:PEI + TAT-pDNA nanocomplex solutions were prepared as follows:

- pDNA (3.5 μg) + βTCPYbTm:PEI (35 μL)- Vortex 3 seconds, spin and incubate at room temperature for 20 min, or
- (pDNA (1.5 μg) + TAT (150 μL)) - Vortex 3 seconds, spin and incubate at room temperature for 20 min. Followed by the addition of βTCPYbTm:PEI (20 μL), inversion of the tube upside down thrice, to gently mix the solution, spin and incubation for an extra 20 min.

After the incubation steps, required for DNA binding, each βTCPYbTm:PEI-pDNA(+TAT) complex dispersions were diluted in 1% FBS-containing DMEM medium (500 μL), and then transferred to the cell culture wells. The cells were incubated for 6 h in a humidified CO_2_ controlled atmosphere before being supplied with extra supplemented fresh medium for a final concentration of 10% FBS-containing DMEM and further cultivation until 24 h post-transfection. Nanocomplexes-containing media were removed, cells were gently rinsed twice with PBS, and slowly supplied with fresh complete DMEM. Reporter gene expression was determined 48 h post-transfection in a Flow Cytometer (Attune NxT).

### Cytotoxicity Assay

HEK293T were cultured in DMEM containing high glucose (4.5 g L^-1^ at 25 mM) and supplemented with penicillin (100 units mL^-1^), Streptomycin (50 mg mL^-1^), and FBS (10%), seeded in 96-well plates at a density of 5x10^3^ cells per well and incubated for 24 h before the assay. The different nanovectors were tested with and without TAT peptide and pDNA, simulating the transfection assays. After cells were exposed to nanocarriers for 24 h, the apoptotic cells were evaluated by 3-(4,5-dimethylthiazol-2-yl)-5-(3-carboxymethoxyphenyl)-2-(4-sulfophenyl)-2H-tetrazolium (MTS) assay, according to the manufacturer's instructions (CellTiter-Glo 96® aqueous one solution cell proliferation kit (Promega-USA)), and absorbance was measured at 490 nm using a microplate reader (Thermo Scientific Multiskan Ex).

### Flow Cytometry

After the transfection assays, cells were washed, harvested, centrifuged and resuspended in FBS (2%) -containing PBS and kept in the dark on ice until analysis. Cellular transfection quantification was evaluated through flow cytometry (Attune NxT) analysis, exciting samples at 488 nm and acquiring emission around 525 nm, for GFP excitation. Cells viability was also quantified by flow cytometer analysis. Live cells were analyzed using 7AAD fluorochrome (Bio-Rad) to stain non-viable cells, exciting cells at 488 nm and emission wavelength were acquired at 647 nm 16.

### Live and fixed cells imaging

Live cell imaging was performed using the Zeiss LSM 880, an inverted laser scanning confocal microscope equipped with high quantum efficiency GaAsP detector and 2 multialkali PMTs, and 6 lasers (Diodo 405 nm, Argon ion 458 nm, 488 nm and 514 nm, DPSS 561 nm, HeNe 594 nm, HeBe 633 nm and Mai Tai Insight DeepSee, (tunable 680-1300 nm, pulse width < 120 s and repetition rate 80 MHz), temperature and CO_2_ control in full enclosure incubator. Image sequences were captured at approximately 30 min intervals, for up to 24 h. Cells were cultivated in Fluorodish plates and nucleus was stained with Hoechst dye, following manufactory's instruction.

Fixed cells images were acquired in conventional inverted fluorescence microscopy or with Leica LSM 880. For this purpose, cells were cultivated in glass coverslips and after the transfection procedure, described as above, cells were washed twice with PBS, fixed with formaldehyde (4%) for 15 min and stained with DAPI.

For the cell-nanoparticle interaction assays, the L929 murine fibroblast cell line was used, following the same culturing protocol described for HEK293T cells.

### Statistical analysis

Results are expressed as the mean ± standard error of the mean (SEM). Statistical significance was determined by analysis of variance (ANOVA) with * p < 0.05 and ** p < 0.01.

## Results and discussion

### Synthesis and Characterization of βTCPYbTm nanoparticles

**Figure 1:**
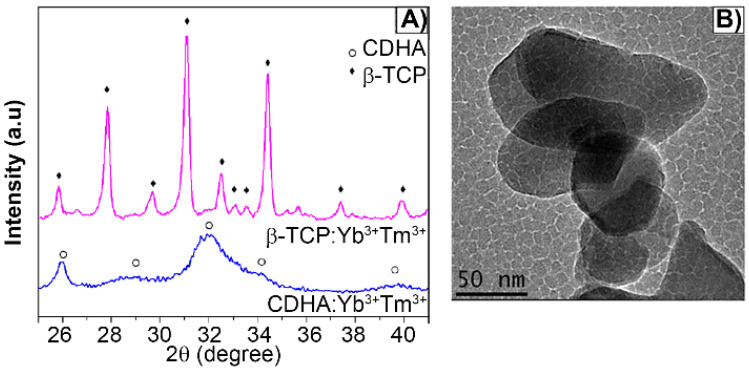
A) X-ray diffraction pattern of Yb/Tm-doped calcium phosphate nanoparticles as synthesized (CDHA phase) and treated at 900 °C for 3 min (β-TCP phase). B) TEM micrograph of bare βTCPYbTm nanoparticles.

The as-synthesized lanthanide-based calcium phosphate nanoparticles, corresponding to ytterbium and thulium-doped calcium-deficient hydroxyapatite (CDHAYbTm), have shown the hexagonal phase HA, corresponding to the JCPDS file nº 9-432, with a broadening diffraction profile, as seen in Figure 1A). After being thermally treated at 900 °C for 3 min, in an adapted domestic microwave oven 8, the powder showed well characterized peaks of β-TCP phase, indexed according to the standard DRX pattern JCPDS file nº 13-404 (Figure 1A). Figure 1B displays the βTCPYbTm nanoparticles morphology, showing an irregular oval or circular shapes and an average size smaller than 100 nm 8.

**Figure 2:**
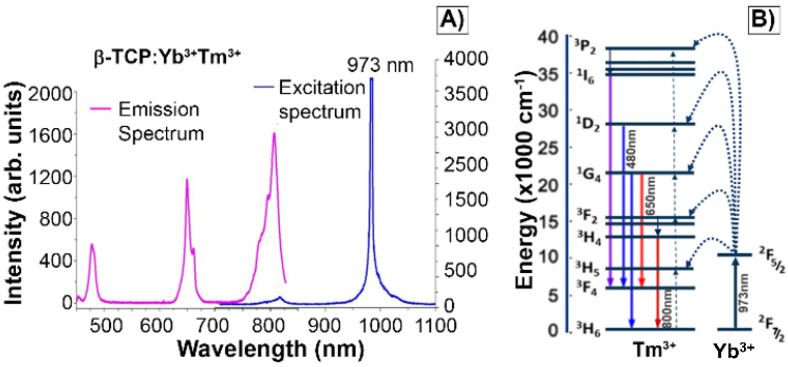
A) Emission and excitation spectra of βTCPYbTm nanoparticles. Emission bands of Tm^3+^ excited by upconversion process through energy transfer from Yb^3+^, using pulsed laser at 973 nm. B) Schematic diagram of energy level transition of Yb^3+^ - Tm^3+^ ions in the upconversion process.

The characteristic excitation and emission spectra of βTCPYbTm (under the 973 nm laser excitation) are given in Figure 2A. The four emission bands centered around 450 nm, 480 nm, 650 nm and 800 nm can be assigned to ^1^D_2_ → ^3^F_4_, ^1^G_4_ → ^3^H_6_, ^1^G_4_ → ^3^F_4_ and ^3^H_4_ → ^3^H_6_ transitions of Tm^3+^, respectively. The populations of the states ^1^D_2_, ^1^G_4_ and ^3^H_4_ come from four-photon, three-photon, and two photon upconversion processes, respectively (Figure 2B). Ytterbium ions act as a sensitizer in the upconversion luminescence process, then the energy is effectively transferred from the excited absorption state of Yb^3+^ to Tm^3+^ ion in calcium phosphate, due to its low phonon energy and high quantum efficiency as luminescent host 8. Regarding the luminescence lifetime, previously discussed by our group 8, the βTCPYbTm nanoparticles have shown a risetime of 37µs (three-photon processes are involved to populate the ^1^G_4_ state) and long decay time constants of 153 and 120 µs, for the blue (^1^G_4_ → ^3^H_6_), and red (^1^G_4_ → ^3^F_4_) emissions, respectively.

### PEI-coated βTCPYbTm nanoparticles

βTCPYbTm was functionalized with PEI to produce positively charged nanoparticles 17,18. A comparative analysis of size and surface charge by DLS was performed for all PEI-coated βTCPYbTm complexes, prepared at pH 5.2 and pH 7.3. The obtained data (Table 1) shows that bare βTCPYbTm in water has a slightly negative zeta potential value, around -2 mV, that were reversed to positive charge for all PEI-coated βTCPYbTm nanoparticles.

PEI concentrations were fixed at 1 mg (in 10 mL of MilliQ water, 4 ng mol^-1^), while βTCPYbTm concentrations ranged from 0.5 to 10 mg (in 10 mL, corresponding to 50 - 1000 ng mol^-1^), for βTCPYbTm/PEI ratios varying from 12.5 to 250:1 w/w. For these conditions, the zeta potential values varied between +23 mV and +47 mV. On the other hand, the size (Z-Average) did not varied considerably at pH 5.2, being around 227-255 nm, whereas for pH 7.3, the smallest Z-Average was observed for 12.5:1 w/w βTCPYbTm:PEI (≈250 nm), with a positive surface charge of 31 mV and a polydispersity index (PDI) around 0.3, being considered as a monodispersed solution.

Additionally, it could be noticed that after purification (centrifugation and washing thrice with deionized water), the complex (50:1 w/w βTCPYbTm:PEI) presented no difference in its size, ≈250 nm, but its zeta potential dropped from +47.10 mV to +25.67 mV. This result suggests that the centrifugation/washing steps during the preparation of the nanocomplexes removes some PEI molecules, thus reducing the net surface positive charge. Measurements were conducted in triplicate and are expressed as mean ± standard error of the mean.

### Cell internalization study

Prior to transfection, the cellular uptake process of both kinds of nanoparticles (bare and PEI-coated) was studied. For this purpose, the upconversion nanoparticles were evaluated at different concentrations in L929 cell line, after 24 or 48 h of incubation.

**Figure 3:**
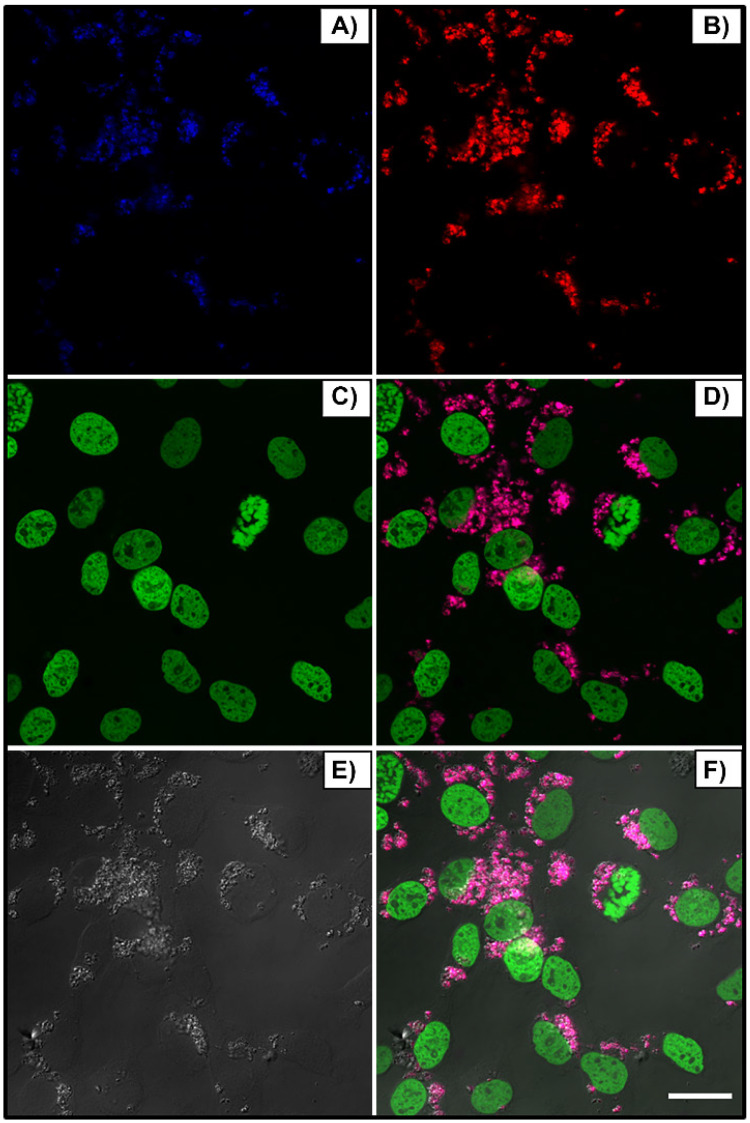
Upconversion nanoparticles internalization by L929 cell line, after 24h of incubation. The blue (A, λ_em_ ≈ 450-480 nm) and red (B, λ_em_ = 630-670 nm) nanoparticle emissions when excited at 975 nm, the nucleus in green (C, λ_ex_ = 488 nm), bright field (E) and merged channels (D and F). Scale bar = 75 μm.

Figure 3 shows the blue (A) and red (B) nanoparticle emissions, peaked around 480nm and 650nm, respectively, when excited at λ_ex_ = 975 nm, the nucleus in green (C, λ_ex_ = 488 nm), bright field (E) and merged channels (D and F). Cells received 150 μL of nanoparticle solution (250:1 w/w, βTCPYbTm:PEI). After 24 h, cells were washed, fixed and the images were acquired using the Leica LSM 880 inverted confocal microscope.

**Figure 4:**
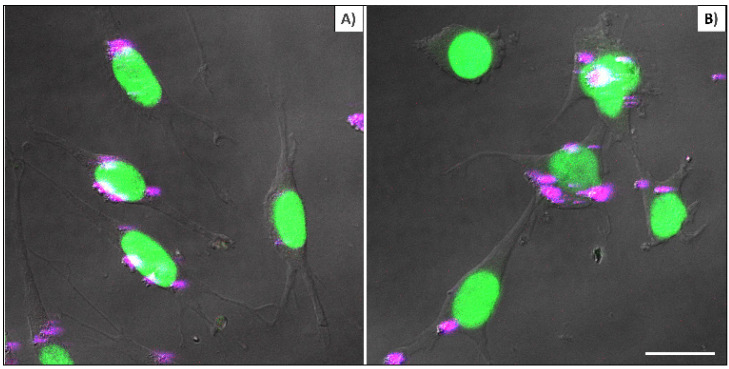
L929 cells cultivated for 48 hours with 50 µL of nanoparticle solutions: (A) 50:1 w/w βTCPYbTm:PEI and B) 50 bare βTCPYbTm. Nanoparticles emission is represented in magenta dots and nuclei emission in green. Scale bar = 75 µm.

Figure 4 compares cellular interaction with PEI-coated (A) and bare (B) βTCPYbTm nanoparticles. Cells were cultivated for 48 h with 50 μL of nanoparticle solutions (50 βTCPYbTm or 50:1 w/w βTCPYbTm:PEI) in 1 mL of complete DMEM, then washed and fixed prior to microscopic analysis. Taken together, these results demonstrated that: 1) bare nanoparticles and those complexed with PEI were taken up by cells and there were no significant differences in their internalization with or without the polymeric coating, meaning that cell-nanoparticles interaction does not require cationic functionalization; 2) large amounts of nanocomplexes can be internalized by cells, confirming the non-cytotoxicity of these lanthanide-based calcium phosphate compounds, even at high concentration; and 3) the upconversion nanoparticles exhibit very bright visible emissions generated through the 975 nm excitation, enabling them to be easily trackable intracellularly, being considered very efficient luminescent probes for cell imaging.

### DNA binding

**Figure 5:**
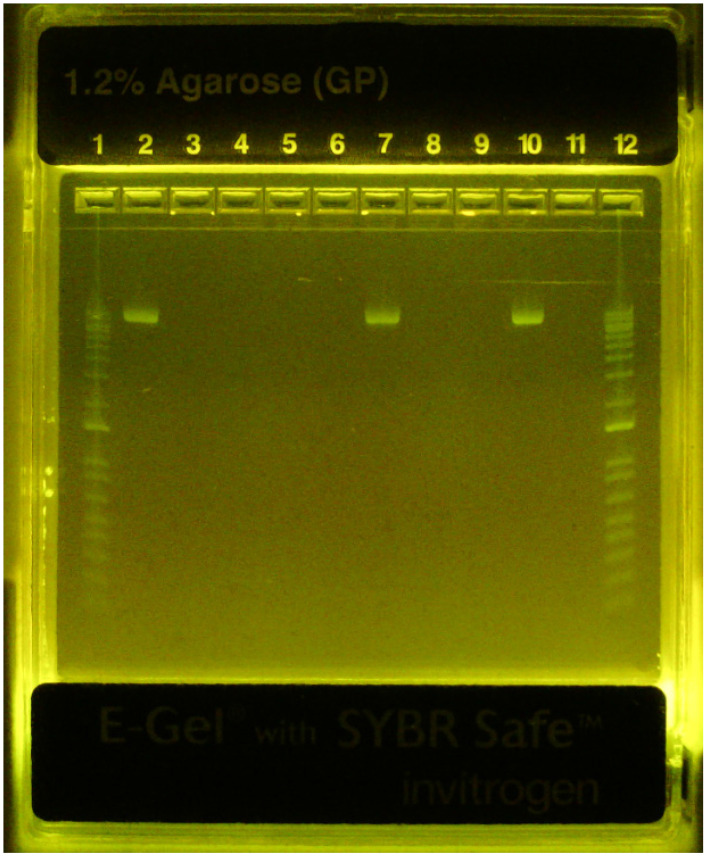
Gel retardation - eGFP-C1 plasmid DNA binding assay. Lane 1: ladder 1000bp; Lane 2: pDNA only; Lane 3: 12.5:1 w/w βTCPYbTm:PEI pH 7.3 + pDNA; Lane 4: 25:1 w/w βTCPYbTm:PEI pH 7.3 + pDNA; Lane 5: 50:1 w/w βTCPYbTm:PEI pH 7.3 + pDNA; Lane 6: 250:1 w/w βTCPYbTm:PEI pH 7.3 + pDNA; Lane 7: 250 bare βTCPYbTm + pDNA; Lane 8: 12.5:1 w/w βTCPYbTm:PEI pH 5.2 + pDNA; Lane 9: 50:1 w/w βTCPYbTm:PEI pH 5.2 + pDNA; Lane 10: 50 bare βTCPYbTm + pDNA; Lane 11: PEI only + pDNA; Lane 12: ladder 1000bp. The pDNA remains unbound only for bare βTCPYbTm suspensions (lanes 7 and 10).

Formation of βTCPYbTm:PEI-pDNA complexes was studied by agarose gel electrophoresis. Figure 5 demonstrates that all PEI-coated βTCPYbTm nanoparticles were able to effectively bind plasmid DNA (eGFP-C1), inferred from the non-appearance of the free plasmid DNA in the relevant lanes. Plasmid DNA retardation was not observed for the bare βTCPYbTm/pDNA ratios studied. The pDNA remained unbound in the bare βTCPYbTm suspensions (lanes 7 and 10), which may be explained by the negatively charged surface of the bare nanoparticles in water, as observed in zeta potential measurements (Table 1), repelling pDNA molecules (also negatively charged).

### Nanocomplex transfection efficiency

The cellular transfection experiments were performed in HEK293T cells using the plasmid encoding the enhanced green fluorescence protein (eGFP) and the expression of GFP induced by the nanocarriers was quantified by flow cytometry analysis and qualitatively examined using fluorescence microscopy.

The overall nanocarriers preparation can be envisioned as having two main stages. The first one involves the coating of calcium phosphate nanoparticles by the polymer. The second part involves the mixing of DNA and nanoplex solution followed by 20 min of incubation to allow complexes to mature, depending on the culturing media conditions.

The first cellular transfection tests revealed higher transfection rates for the non-purified samples in comparison to the centrifuged and washed one (data not shown). The excess of PEI is important for the DNA condensation through the complex electrostatic interactions with the negative charged phosphate backbone of nucleic acid molecules; and the net positive charge facilitates their posterior interaction and fusion with negatively charged lipid cell membranes/surfaces and to be taken up via endocytosis. The high density of positive charges attributed to amine groups provides the 'proton-sponge' effect inside the endosome, which leads to eventual rupture of the endosomal membrane associated with higher probability of endosome escape 19 and, consequently, further releasing of the endocytosed packaging cargo into the cytoplasm 20.

Therefore, all transfection assays were performed with no purification step. To determine the optimal transfection efficiency of the gene non-viral vectors, different transfection incubation times, charge ratios (negative/positive; N/P) and the nanocomplexes-to-pDNA ratios that gives the highest level of transfected cells were investigated. For these experiments, a low amount of pDNA was chosen, in HEK293T cells cultivated in antibiotic and serum free-media 14, in order to enhance the influence of each nanocarrier component and/or variation in the transfection assays. In this approach, 600 ng of pDNA (eGFP-C1) complexed with different volumes of βTCPYbTm/PEI molar ratios varying from 0 to 250:1 w/w resulted in the best configuration for 10 μL of 12.5:1 w/w βTCPYbTm:PEI (18% transfected cells), in comparison to any other CaP/PEI ratios or 10 μL of PEI only (11% transfected cells). For this reason, the molar ratio of 12.5 was chosen to be used on the subsequent experiments, and hereafter it is referred as βTCPYbTm:PEI.

To improve the transfection efficiency, a nuclear targeting peptide, TAT, was added to form a new nanocomplex, comprising the peptide, the luminescent calcium phosphate nanoparticles and the polymer, in order to take advantage of each individual benefit from the different molecules in one single nanocarrier for the plasmid DNA delivery. For this approach, the order of the complex formation directly influenced the transfection rate: a) TAT peptide alone with plasmid DNA did not transfect any cells, which is probably related to TAT's limited capacity for binding DNA, DNA protection and/or incomplete DNA release from endosomal vesicles; b) when TAT is bound to the plasmid DNA and further complexed with βTCPYbTm:PEI or PEI only, the TAT peptide was able to greatly increase the nanocomplexes transfection capability (≈30%), consolidating its role in the translocation of the plasmid DNA into the nucleus of the cells; and c) it is noteworthy when TAT is added after βTCPYbTm:PEI + pDNA complexation, its presence makes absolutely no difference in the final number of transfected cells, probably because the positively charged surface of the complexes do not allow TAT to bind to them; the TAT peptide then remains free in the solution.

Further variation around TAT, nanoparticle:PEI and pDNA solutions revealed that the optimal configuration was 50 μL, 10 μL and 600 ng, respectively, representing around 30% transfected cells. Then, the amount of each component of the system was increased threefold to achieve an improvement in the transfection efficiency rate. However, the number of transfected cells increased only from ≈ 30% to ≈ 40%.

**Figure 6:**
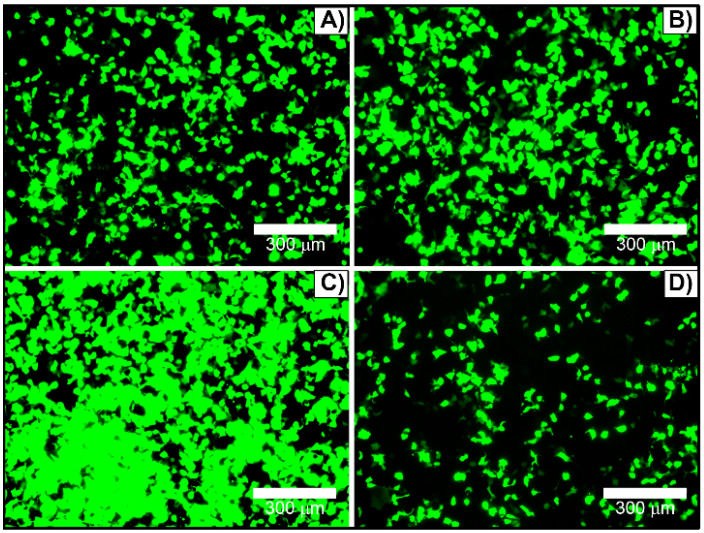
HEK293T cells transfected with [(50 µL TAT + 600 ng pDNA) + 10 µL βTCPYbTm:PEI] in DMEM medium containing: A) free-antibiotic (0% penicillin and streptomycin) and -serum (0% FBS); B) 1% penicillin and streptomycin and 0% FBS; C) 1% penicillin and streptomycin and 1% FBS; and D) 1% penicillin and streptomycin and 10% FBS. Scale bar = 300 μm.

To optimize transfection efficacy, culturing cell parameters have been assessed. Transfections were performed in absence or presence of antibiotics (penicillin and streptomycin) and/or FBS. HEK293T cells could be transfected in growth medium supplemented with 1% penicillin and streptomycin without a markedly reduction in the reporter gene expression, Figure 6A versus 6B. In contrast, serum protein in growth medium can interact with complexes to form larger or smaller aggregates 16 and interfere in the transfection efficiency. The amount of serum-containing DMEM on HEK293T cells transfection was varied between 0% and 10% FBS-containing DMEM media. Surprisingly, transfection performed in the presence of 1% FBS (Figure 6C) leaded to the highest transfection rate, considerably better in comparison with either serum-free (Figure 6A and 6B) or complete supplemented medium (Figure 6D). In fact, the addition of 10% FBS significantly reduced the nanocomplex transfection efficiency (Figure 6D). Additionally, 2% and 3% FBS-containing DMEM were also tested, and 2% FBS did not show significant difference from 1% FBS, while 3% decreased transfection efficiency (data not shown). For this reason, all further transfection assays were performed in 1% penicillin and streptomycin and 1% FBS-containing culturing media.

To understand the role of FBS in the system, DLS analysis were carried out and the results are presented in Table 2. Firstly, FBS-containing DMEM media have an intrinsic negative zeta potential, due to the presence of their negative components 21 and, consequently, the presence of serum in DMEM clearly affected the zeta potential of all vectors studied herein (-7.9±0.4 to -10.1±0.6 mV). Naked plasmid DNA in DMEM (1% FBS) showed the highest negative value (-24.3±0.7 mV) as expected with the negative charge on DNA. A mixture of TAT with the plasmid DNA (pDNA) was slightly less negative with an average zeta potential of -17.5±0.9 mV, indicating that the nucleic acid molecules in the TAT-pDNA complex were exposed on the surface of the peptide-DNA assembly. This means that the plasmid DNA would still be prone to rapid degradation or premature release in the TAT-pDNA complex, which probably dictates the unsuccessful transfection of that complex.

When complexed with βTCPYbTm:PEI or PEI only, the pDNA is fully protected by the nanocomplex (zeta potentials in 1% FBS -DMEM = -6.8±0.2 and -6.0±0.2, respectively). The remaining overall negative charge in these complexes is attributed to the proteins from the media 16 which are adsorbed and alter the surface of the complexes, reversing their surface charge from +30 mV to - 6 mV (free-DNA). Interestingly, the presence of these proteins also leads to a significant rise in dimensions (Z-average) of the complexes, from ≈ 230 nm to ≈ 480 nm, due to the formation of PEI-coated βTCPYbTm nanoparticles agglomeration, followed by protein interactions.

When the pDNA is bound to the complex, in 1% FBS-DMEM, the average diameter of the βTCPYbTm:PEI+pDNA is larger than that in the absence of pDNA, ≈ 530 nm and ≈ 480 nm, respectively (Table 2), indicating that the bigger version contains a number of plasmid bound to the nanocomplexes. Nanocarrier-pDNA complexes are formed by self-assembly through electrostatic interaction between the nanoparticles-cationic compounds and the anionic nucleic acid. However, unexpectedly, when the formed complexes were added to 10% FBS-DMEM, the size of these complexes greatly reduced from > 500 nm to < 160 nm (Table 2). Calcium phosphate nanoparticles are known to be good adsorbents, with high surface area 22,23 that leads to an enhanced capacity of adsorbing proteins from the media. Due to stronger electrostatic interactions, the protein adsorption capacity increases with higher amounts of serum proteins that may then break down the agglomerates, forming smaller nanocomplexes. The presented results showed that FBS played an important role in the size and surface charge of nanoparticles:PEI + pDNA vectors by changing agglomeration configuration and, consequently, the transfection efficacy.

The dimension of the agglomerates is an important parameter governing particle sedimentation. Herein, cells have grown as a monolayer on the surface of the tissue culture flask, and the nanoparticles need to precipitate onto the cells and fuse/interact with cell membrane for gene therapy to take place. The smallest nanoparticles (in 10 % FBS) may remain well dispersed in the media, limiting their ability to interact with cellular receptors/membrane, hindering their efficiency. Freely floating dispersed nanocarriers could be a better transfection strategy for gene edition in suspended cells and for *in vivo* applications.

Nevertheless, low amounts of serum, 1% FBS, lead to the highest transfection rate by providing a way to modify nanocomplexes (larger agglomerates formation) and presumably due to its positive effect on cellular functions (instead of serum-free), which enhances the gene transfection expression.

**Figure 7:**
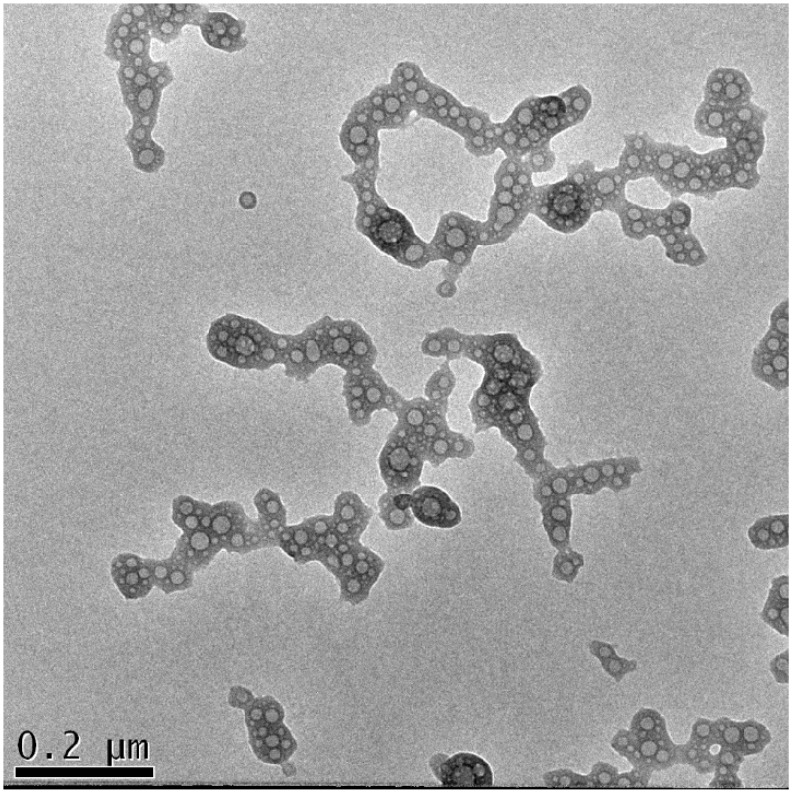
TEM analysis of [βTCPYbTm:PEI + (DNA + TAT)]. Scale bar = 200 nm

Figure 7 shows the morphology of the nanocomplexes prepared with the best transfection system: βTCPYbTm:PEI + (pDNA + TAT) in DMEM containing 1% FBS. The image demonstrates that the plasmid DNA (pDNA) pre-incubated with TAT peptide forms globular vesicles that are incorporated onto the PEI-coated βTCPYbTm, producing irregular clusters on average size around 530 nm (Table 2) that enable them to precipitate, be internalized by cells and efficiently translated into transfected cells.

Following the optimization of the protocol, culturing conditions were evaluated and it was confirmed that after 6 h of cell incubation post-transfection in 1% FBS-DMEM, non-internalized pDNA remained encapsulated and protected in the nanocomplexes, and therefore, viable (data not shown). With this discovery, instead of replacing the media containing the transfection agents 6 h post-transfection, cells were supplied with extra supplemented fresh medium for a final concentration of 10% FBS-DMEM, without removing the nanocomplexes-containing media, for further cultivation in humidified atmosphere at 37 °C. After a total of 24 h, media were removed, cells were gently rinsed twice with PBS, and slowly supplied with fresh complete DMEM. Reporter gene expression was determined 48 h post-transfection by flow cytometer or fluorescence microscopy.

**Figure 8:**
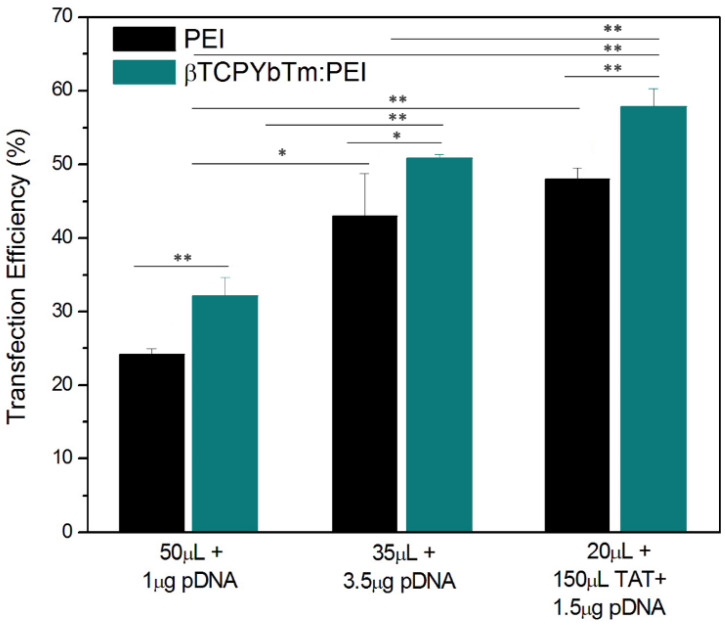
Transfection efficiency of PEI only versus βTCPYbTm:PEI nanocarriers, showing that lanthanide-based calcium phosphate nanoparticles, in the presence or absence of TAT peptide, improved the percentage of transfected cells; *p < 0.05 and ** p < 0.01.

Once the optimized condition was determined, the amounts of pDNA and βTCPYbTm:PEI versus PEI only were varied again, and for the improved protocol, it could be noticed that there were no differences in the percentage of transfected cells between the negative/positive (N/P) charge ratio = 10, usually used in literature 16 and references in 16 (1 μg pDNA + 50 μL Nanocomplex) or N/P ≈ 2 (1 μg pDNA + 15 μL Nanocomplex). In both cases, for PEI only, transfected cells were around 25%, whilst for βTCPYbTm:PEI the percentage was around 33%. Reducing the amount of PEI reagent required for the transfection, using N/P ≈ 2 (instead of the usual 10), induces less toxicity for cells, and increases cell viability. In addition, for 35 μL βTCPYbTm:PEI + 3.5μg pDNA, it reached the highest number of transfected cells: around 50 % to βTCPYbTm:PEI, against 40% to PEI only. Experiments were conducted in at least quadruplicate, in independent experiments, and are expressed as mean ± standard error of the mean in Figure 8. The values of 50% and 40% of transfected cells, with and without nanoparticles, respectively, were considerably higher than those conventionally reported in papers for HEK293T cells using PEI as nonviral carrier: 22-35% 24-26.

The expression of GFP increased along with the increasing of nucleic acid amount complexed with CaP:PEI up to 3.5 μg of pDNA, then remained the same or even decreased with higher concentrations due to the cytotoxicity related with the large amounts of polymer and/or exogenous DNA and cells death.

Nevertheless, it was possible to achieve highest transfection rates with the addition of TAT peptide. The cytometric analysis revealed that the transfection efficiency reached up to ≈ 60% for the best working condition of [(1.5 μg pDNA + 150 μL TAT) + 20 μL βTCPYbTm:PEI]. Any other pDNA, TAT or βTCPYbTm:PEI concentrations/ratios did not improve the number of transfected cells. Addition of plasmid DNA at amounts higher than the optimal DNA:PEI ratio was not translated into more transfected cells 27. It appears that successful transgene expression is dependent not only on the number of plasmids internalized and delivered per cells, but on the number of plasmids translocated into the cell's nuclei, and also on the cellular capacity or machinery transcriptional competency to result in the translation of the protein and its accumulation in the cytoplasm of the cell 6. In addition, the detection limits of fluorescent technique/equipment must be taken into account, while it is not possible to know whether the 'negative cells' are transfected cells expressing activity below the sensitivity of the flow cytometry quantification or whether the remaining cells are truly negative.

Transfection efficiency of the complexes composed by lanthanide-based calcium nanoparticles, PEI and TAT is closely tied to nucleic acid binding, the packaging released in the cytoplasm, the dissociation of the payload from the complex, and the translocation of viable nucleic acid cargo to the nucleus for the subsequent transcription and translation processes. In this context, each component in the nanocomplex has its own role in the transfection events: PEI binds and condenses DNA efficiently, providing their cellular uptake and endosomal releasing through the 'proton-sponge' effect in which the absorption of proton (H^+^) by the free amine groups induces the osmotic swelling event leading to the endosome membrane rupture 28. Calcium phosphate nanoparticles reduce PEI cytotoxicity, and once the nanocomplexes are released into the cytosol, calcium ions may provide a balance between DNA binding affinity and ease the dissociation of the payload from PEI, stabilizing and protecting DNA from intracellular degradation. The stabilization of the plasmid by Ca^2+^ has been associated with the plasmid being released in the supercoiled form 29. Escape from the endolysolitic pathway is not enough to ensure high transfection efficiency. Once free in cytosolic environment, DNA must reach the nucleus to expression occurs. Calcium ions are reported to facilitate nucleic acid transportation through nuclear pore complexes (NPC) located throughout the nuclear envelope, i.e. the double membrane or boundary between the nucleus and the cytoplasm of the cell, which serves as a selective barrier by actively transporting molecules required for cell function, as well as acting as a barrier to the entry for free nucleic acid from the cytoplasm 24. Ca^2+^ modulates the passage and transport of proteins and nucleic acids through NPC 24, suggesting an NPC plug associated with Ca^2+^ ion-dependent mechanism. Under such circumstances, free Ca^2+^ dissociated from the high solubility/dissolution βTCPYbTm nanoparticles forms DNA-Ca^2+^ complexes that can easily translocate the nuclear barrier through the NPC by calcium ion- assisted conformation and function of NPC 24. The second known role, and perhaps the most widespread mechanism for polymer-mediated gene delivery 23, for DNA transport to the nucleus, is via association with chromatin during mitosis/cell division. Once within the nucleus, DNA is transcribed into mRNA, and subsequently translated into a protein, which is the targeted gene product.

It has been reported that a minimum number of 10^2^-10^4^ plasmid copies in the nucleus is required for transgene expression be detectable 23 and that cells with up to 550 copies of GFP plasmids were sorted as negative cells 22. Interestingly, they reported that analyses performed on sorted cells revealed that negative and positive cells had equal amounts of plasmid DNA, however, the plasmid content in the nucleus of the eGPF-positive populations was three-fold higher (1850 copies) than in -negative cells (550 copies) 22. In this context, the addition of CaP nanoparticles is advantageous over the PEI only as nonviral vectors in the sense that they successfully overcome nuclear translocation limitations, delivering the plasmids to the nucleus, and enhance transfection efficiency.

Nucleocytoplasmic transport through the NPC may occur by the assistance of Ca^2+^- regulated transport, as above described, or through active transport, with the support of nuclear localization signals (NLS) 25. NLS are sequences of highly cationic peptides explored for cellular import, mediated by an energy-dependent translocation via recognition of NLS and subsequent release of cargo into the nucleus through NPC 30. The TAT peptide is one of these peptide sequences identified with cell-translocating properties, or more specifically as nuclear targeting peptide, which can be covalently or noncovalently attached to nucleic acid 25. The latter is the approach employed in this work, in which TAT peptide was electrostatically complexed with plasmid DNA, taking one step further by targeting specifically the nucleus of the cells. By increasing the proportion of active pDNA accumulated in the subcellular target site of action inside the cell, a reduced plasmid concentration was required for a higher transfection rate. Combining all those aforementioned advantages of each component in all-one-nanocomplex allowed the system to get the highest efficient rate of more than 60% transfected cells.

Collectively, the results suggest that, not only the delivery of plasmids by the nonviral vectors to cells, but the processes governing the nuclear transportation and further processing of the nucleic acid as well were important for the efficient expression. Therefore, gene expression is not necessarily completely proportional to the amount of plasmids delivered into the nucleus, but shows saturation when an excess over optimal quantity of DNA is delivered into the nuclear compartment 31. Once saturated, the capacity of gene transcription appears to be primarily a result of the inability of those transfected cells to translate the gene into protein. Thus, it seems that successful transgene expression depends on of a combination of nonviral gene delivery system overcoming the numerous chemical, physical and biological barriers encountered between the nanocomplex administration and plasmid localization in the cell nucleus, and of cellular transcriptional/translational competency and/or limitations 22.

### Cell viability

*In vitro* cytotoxicity of the nanovectors were assessed using the MTS assay and live/dead assay with 7AAD by flow cytometry analysis to test the relative cell viability of HEK293T cells incubated with PEI only, bare βTCPYbTm, βTCPYbTm:PEI, TAT only and pDNA loaded PEI, βTCPYbTm:PEI with and without TAT peptide. The ratios of pDNA, nanovectors and TAT were fixed as the most efficient ratios on the previous experiments (transfection efficiency).

**Figure 9:**
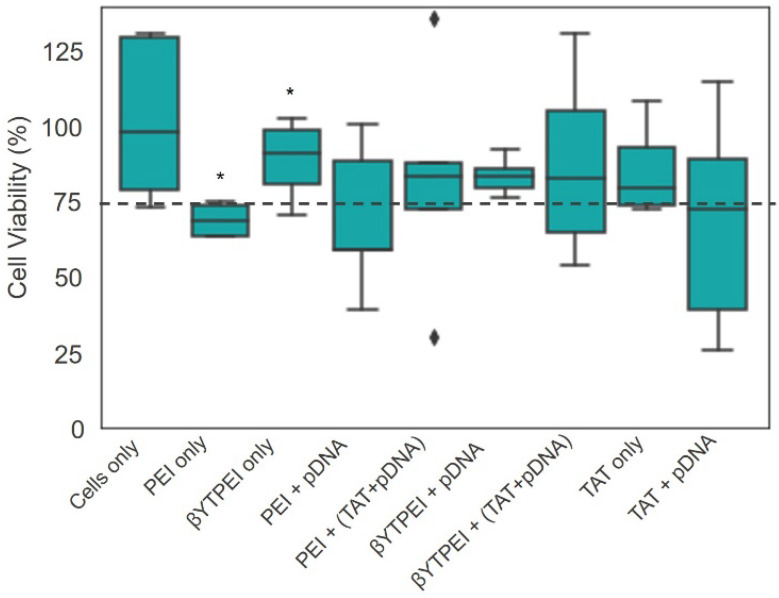
Cell viability test. For the cytotoxicity evaluation, using the MTS assay, the HEK293T cells were incubated for 24 hours with the nanocarriers in the presence and absence of the pDNA (* p < 0.05). (βYTPEI abbreviation stands for βTCPYbTm:PEI).

By flow cytometry analysis (7AAD live/dead assay) and 3-(4,5-dimethylthiazol-2-yl)-5-(3-carboxymethoxyphenyl)-2-(4-sulfophenyl)-2H-tetrazolium (MTS) assay (Figure 9), cells when treated with the different nanocomplexes and determined, concomitantly, with gene expression, showed that the presence of βTCPYbTm has decreased PEI cytotoxicity (live cells), and did not show significant statistically difference on the number of live cells in comparison with control group. Only βTCPYbTm:PEI showed statistically significant difference against PEI only, and no further significant differences were observed between the treated and control cells (no treated cells), reconfirming the good biocompatibility of the nanocomplexes. Again, TAT + pDNA showed the lower cell viable percentage (around 75%), and the presence of the nanocomplexes decreased its intrinsic cytotoxicity. Experiments were conducted in quintuplicate and are expressed as mean ± standard deviation of the mean.

In general, the cytotoxicity induced by cationic polymers, such as PEI, increases along with their charge density 6, and once PEI is incorporating the calcium phosphate nanoparticles, less of the cationic residues of PEI are able to interact with the cells, decreasing its cytotoxicity 6. The addition of TAT increased the cytotoxicity of the nanocomplexes and, more significantly, TAT alone with pDNA presented the lower cell viability. For these measurements, GFP and 7-AAD fluorochromes were excited at 488 nm and emissions were acquired at 525 nm and 647 nm, respectively.

### Upconversion Nanoparticle for image-guided gene therapy

Lanthanide-based upconversion nanoparticles have become attractive for biomedical applications by virtue of their unique optical properties and multi-modality imaging ability. More importantly, herein they were used as a multi-functional biomaterial for image-guided gene therapy, when the lanthanide-based upconversion nanoparticles are complexed with plasmid DNA, acting as a gene carrier as well as a fluorescent probe, simultaneously.

Triple lanthanide-based beta tricalcium phosphate nanoplexes were successfully produced as a new promising class of lanthanide-based upconversion nanoparticles functionalized with PEI and conjugated with the nuclear targeting peptide (TAT) electrostatically attached to plasmid DNA. The usage of this ternary nanocomplex as theranostic tool combines transfection carrier properties for the plasmid DNA, allowing the endocytosis-mediated transport of the nucleic acid across the cell and nucleus membranes, with the nanoparticle tracking through their very bright intrinsic fluorescence as imaging probes to analyze nonviral vector-cell interactions.

**Figure 10:**
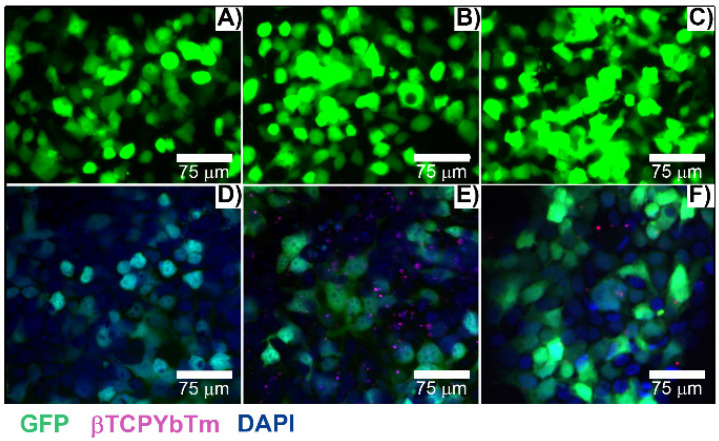
Transfected cells with A/D) 35 µL of PEI only + 3.5 µg pDNA; B/E) 35 µL of βTCPYbTm:PEI + 3.5 µg pDNA and C/F) [20 µL βTCPYbTm + (150 µL TAT + 1.5 µg pDNA)], imaged 48 h post-transfected. Green emission represents GFP (λ_ex_ = 488 nm); magenta dots are the upconversion nanoparticles (λ_ex_ = 975 nm) and blue emission are the nucleus stained with DAPI (λ_ex_ = 405 nm). Scale bar = 75 µm.

Due to their luminescent characteristics, a confocal multiphoton microscope was used to observe the nanoparticles internalization by cells 32 and results are shown in Figure 10. The number of transfected cells is consistent with the flow cytometry quantitative analysis (see Fig. 8). Green emission represents the positive transfected cells (excitation at 488 nm), blue emission (Figure 10D-10F) shows the nuclei of the cells stained with DAPI (λ_ex_ = 405 nm), and the magenta dots (Figure 10E and 10F) are the fluorescent upconversion nanoparticles, excited at 975 nm. Figure 10A-10C) represent the conventional fluorescence micrographs for the same cells.

**Figure 11:**
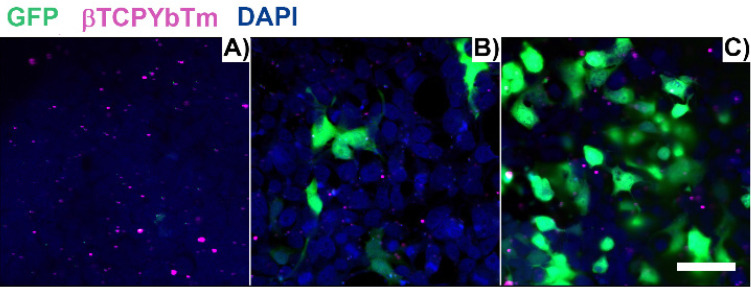
HEK293T cells transfected with [20µL βTCPYbTm + (150 µL TAT + 1.5 µg pDNA)], imaged 1 h (A), 6 h (B) and 24 h (C) post-transfection. Green emission represents GFP (λ_ex_ = 488 nm); magenta dots are the upconversion nanoparticles (λ_ex_ = 975 nm) and blue emission are the nucleus stained with DAPI (λ_ex_ = 405nm). Scale bar = 75 µm.

Figure 11 shows the fixed HEK293T cells one (A), six (B) and twenty four (C) hours post transfection, with [(1.5 μg de pDNA + 150 μL TAT) + 20 μL de βTCPYbTm:PEI]. After 1 h, transfection has already commenced and one green area is spotted at Figure 11.A), in the visualized region in the image. With 6 h, a larger number of cells (less than 10 in the analyzed area), with brighter emitting cells is observed in Figure 11B, and this number is greater in Figure 11C, 24 h post transfection, representing at least 50% of transfected cells (green emission). Cells nuclei are labeled with DAPI (blue emission) and βTCPYbTm:PEI nanoparticles emission is shown as the magenta dots.

**Figure 12:**
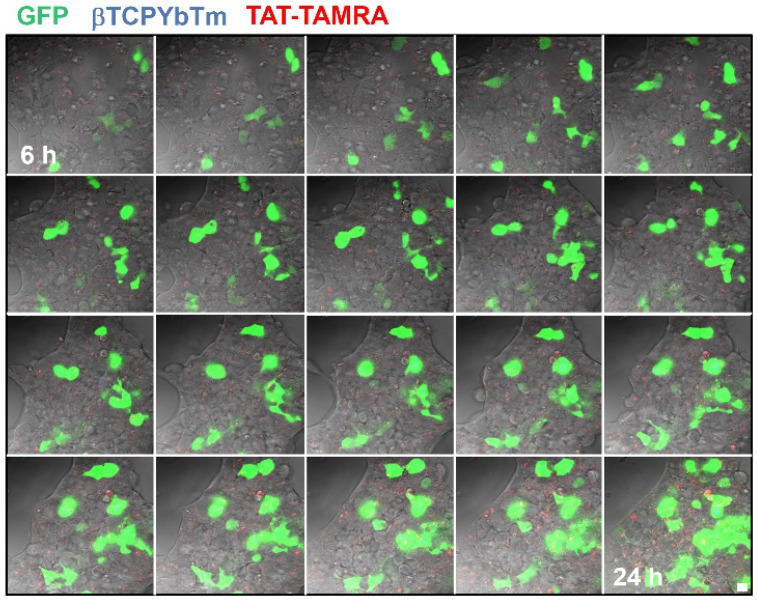
HEK293T cells transfected with [20µL βTCPYbTm:PEI + (150 µL TAT-TAMRA + 1.5 µg eGFP] for overnight analysis from 6 to 24 h post-transfection. Images were acquired every 30 min by the LSM 880 multiphoton inverted confocal microscope. Samples were excited at λ_ex_ = 975 nm (blue emission - nanoparticles), λ_ex_ = 488 nm (green emission - GFP) and λ_ex_ = 561 nm (red emission -TAT-TAMRA). Scale bar = 75 µm.

In a time lapse overnight analysis (Figure 12) it is demonstrated that not only the number of cells emitting the GFP increases as well as the fluorescence intensity of each individual fluorescent cell due to the larger number of the green fluorescent protein produced/secreted during the period analyzed. Six hours after the addition of the nanocomplexes, the green emission is weaker and observed only in few cells (first image frame of Figure 12). As the gene transfection proceeds, the green fluorescence intensity gradually increases along with the number of cells emitting the GFP, reflecting the gradual internalization, release, dissociation and nuclear translocation of pDNA from the [βTCPYbTm:PEI + (TAT-TAMRA + eGFP)] nanocomplexes and further translation into protein. It is worth noting that after 24 h (last frame of Figure 12), the green emission intensity is stronger and continues to increase. Figure 12 represents cells tracked from 6 to 24 h post-transfection, in which the green color indicates the GFP emission (λ_ex_ = 488 nm), nanoparticles as blue dots (λ_ex_ = 975nm) and TAT-TAMRA emission in red color (λ_ex_ = 561nm), merged with the bright field channel.

It was not possible to confirm whether TAT-TAMRA peptide was able to reach the cell nuclei or not. However, it seems that TAT-TAMRA is also in the cytoplasm of the cells (not inside the nuclei), and although TAT is a nuclear targeting peptide and it proved improving gene transfection, when it was labeled with TAMRA, the fluorescent marker could alter its properties, making it difficult to penetrate the cell nuclear membrane 33.

## Conclusions

In summary, this work comprises the development and characterization of a novel nanocomplex composed by an upconversion theranostic lanthanide-based calcium phosphate nanoparticle/polymer/peptide tertiary system capable of efficiently delivering nucleic acids into cells, and have combined it with multi-functional imaging properties as a trackable probe for biological applications.

The nanocomplexes have shown a great potential as image-guided gene carrier for biological use, exhibiting high transfection efficiency, enhanced upconversion fluorescence emission, allowing the observation of the cell internalization process and intracellular fate of nanoparticles, using high resolution fluorescence techniques and low cytotoxicity *in vitro*.

Owing to their variety of appealing properties, calcium phosphates have been considered one of the most promising inorganic non-viral vector for gene therapy. Herein, it was reported on the optimization of transfection conditions and improvement of transfection efficiency by the assembly of βTCPYbTm nanoparticles, PEI, TAT and pDNA, in which PEI is responsible to bind and condense DNA, to provide the nanocomplexes uptaking and to their release into the cytoplasm of the cells. βTCPYbTm plays an important role in assurance the balance between DNA binding affinity and an ease dissociation of the payload from PEI, stabilizing and protecting DNA from intracellular degradation. The free Ca^2+^ ions dissociated from the high solubility/dissolution of βTCPYbTm nanoparticles forms DNA-Ca^2+^ complexes that can easily translocate the nuclear barrier through the NPC by calcium ion- assisted conformation and function of NPC. In its turn, TAT peptide, as a nuclear targeting peptide, was noncovalently attached to nucleic acid and then electrostatically complexed with βTCPYbTm:PEI, taking one step further by increasing the proportion of active DNA accumulated in the nucleus of the cell. Combining all those aforementioned advantageous of each component in all-one-nanocomplex with an optimized protocol allowed the system to achieve efficient rates as high as 60% of transfected cells.

Another advancement in the design of delivery system is the combination of target imaging by optical techniques. Molecular imaging methods such as image-guided technique is playing an important role in biotechnology research. The application of these combined systems allows for target imaging, early detection and the possibility of concomitant treatment of diseases by drug and gene delivery, including vaccines. The facile fabrication process, advantages in terms of ease of large-scale production, simplicity of further modification (surface functionalization) and great biocompatibility, enhanced gene transfection efficiency and great bioimaging ability can make it promising for application in guided-gene therapy, offering promising ways to prepare a suitable system for future clinical use. In addition, one can take advantage of the βTCPYbTm strong emissions for gene light-controlled releasing, by using a light sensitive polymer functionalization approaches.

Bringing together all outcomes of this study, it can be concluded that the usage of lanthanide-based beta tricalcium phosphate nanoparticles was demonstrated as an effective and valuable system that will give rise to versatile useful biomedical and molecular biology relevant theranostic tools.

## Figures and Tables

**Table 1:** A comparative analysis of size and surface charge by DLS analysis of bare and all PEI-coated βTCPYbTm complexes, prepared at pH 5.2 and pH 7.3.

	Zeta Potential [mV]	Size (Z-Average) [nm]	PDI
12.5:1 w/w βTCPYbTm : PEI (pH 7.3)	+31.0 ± 2.1	249 ± 9	0.30 ± 0.05
25:1 w/w βTCPYbTm : PEI (pH 7.3)	+43.5 ± 0.2	267 ± 24	0.28 ± 0.03
50:1 w/w βTCPYbTm : PEI (pH 7.3)	+44.0 ± 0.5	455 ± 21	0.39 ± 0.04
125:1 w/w βTCPYbTm : PEI (pH 7.3)	+44.6 ± 0.8	280 ± 3	0.26 ± 0.02
12.5:1 w/w βTCPYbTm : PEI (pH 5.2)	+23.0 ± 0.1	227 ± 14	0.26 ± 0.03
25:1 w/w βTCPYbTm : PEI (pH 5.2)	+26.1 ± 0.1	255 ± 27	0.32 ± 0.07
50:1 w/w βTCPYbTm : PEI (pH 5.2)	+47.1 ± 0.4	250 ± 13	0.33 ± 0.02
125:1 w/w βTCPYbTm : PEI (pH 5.2)	+42.7 ± 1.2	244 ± 4	0.25 ± 0.01
250:1 w/w βTCPYbTm : PEI (pH 5.2)	+38.9 ± 0.4	254 ± 6	0.26 ± 0.04
50:1 w/w βTCPYbTm:PEI(pH5.2) CW^a)^	+25.7 ± 0.3	242 ± 9	0.29 ± 0.03
50 bare βTCPYbTm	-2.0 ± 0.4	384 ± 40	0.39 ± 0.06
PEI only (pH 7.3)	+5.6 ± 4.4	147 ± 83	0.53 ± 0.09

^a)^CW - Centrifuged and washed thrice with deionized water.

**Table 2:** DLS analysis of the nanocomplexes before and after being loaded with pDNA, for the transfection assay in the presence of 1% or 10% FBS- DMEM. Measurements were conducted in triplicate and are expressed as mean ± standard error of the mean.

	Zeta Potential [mV]	Size Z-Average [nm]	PDI
DMEM 1% FBS	-10.1 ± 0.6		
DMEM 10%FBS	-7.9 ± 0.4		
DNA in DMEM 1% FBS	-24.3 ± 0.7		
(TAT + pDNA) in DMEM 1% FBS	-17.5 ± 0.9		
βTCPYbTm:PEI in DMEM 1% FBS	-6.8 ± 0.2	474 ± 9	0.37 ± 0.01
(βTCPYbTm:PEI+pDNA) in DMEM 1% FBS	-4.4 ± 0.2	527 ± 23	0.41 ± 0.05
(βTCPYbTm:PEI+pDNA) in DMEM 10%FBS	-3.5 ± 0.2	159 ± 3	0.39 ± 0.04
(PEI only + pDNA) in DMEM 1% FBS	-6.0 ± 0.2	456 ± 14	0.46 ± 0.02
